# Glucagon-like peptide-1 receptor agonists in patients treated with antipsychotics

**DOI:** 10.1192/j.eurpsy.2021.2050

**Published:** 2021-08-13

**Authors:** A. Delgado, J. Velosa, R. Avelar, J. Franco, M. Heitor

**Affiliations:** Psychiatry, Hospital Beatriz Angelo, Loures, Portugal

**Keywords:** GLP-1RA, glucagon like peptide, obesity, Antipsychotics

## Abstract

**Introduction:**

Glucagon-like peptide-1 (GLP-1) is an endogenous peptide that stimulates insulin secretion and decreases glucagon secretion. The use of GLP-1 receptor agonists (GLP-1RA) showed efficacy reducing the weight and glucose levels in patients with and without type 2 diabetes. This effect was also associated with a decreased risk of major cardiovascular events.

**Objectives:**

Our aim is to review the role of GLP-1RA in psychiatric patients at cardio-metabolic risk due to antipsychotics treatment.

**Methods:**

We reviewed articles published in PubMed using the keywords: “GLP-1” “glucagon like peptide” “antipsychotics” and “psychiatry”.

**Results:**

The number need to treat (NNT) to achieve clinical meaningful weight loss was 3.8. GLP-1RA treatment was also associated with greater reductions in body mass index, fasting glucose, HbA1c and visceral fat. This effect is true for antipsychotic treatment in general and for those on clozapine and olanzapine in particular. Overall, the GLP-1RA are well tolerated with nausea being the most common related adverse effect. Other variables such as age, sex, psychosis severity, nausea or any adverse drug reaction did not affect the weight loss.

**Conclusions:**

Studies showed a promising role in the management of antipsychotics induced weight gain, particularly in clozapine and olanzapine treated patients. Although these promising results, the route of administration, with a daily or weekly subcutaneous injection, and the GLP-1RA associated financial costs, can be viewed as important factors which can limit the wide use of this type of treatment in psychiatric patients.

**Disclosure:**

No significant relationships.

